# The impact of different strategies for modeling associations between medications at low doses and health outcomes: a simulation study and practical application to postpartum opioid use

**DOI:** 10.1093/aje/kwae147

**Published:** 2024-06-21

**Authors:** Andrew J Spieker, Margaret A Adgent, Sarah S Osmundson, Sharon E Phillips, Ed Mitchel Jr, Ashley A Leech, Carlos G Grijalva, Andrew D Wiese

**Affiliations:** Department of Biostatistics, Vanderbilt University Medical Center, Nashville, TN 37203, United States; Department of Health Policy, Vanderbilt University Medical Center, Nashville, TN 37203, United States; Department of Obstetrics and Gynecology, Vanderbilt University Medical Center, Nashville, TN 37232, United States; Department of Biostatistics, Vanderbilt University Medical Center, Nashville, TN 37203, United States; Department of Health Policy, Vanderbilt University Medical Center, Nashville, TN 37203, United States; Department of Health Policy, Vanderbilt University Medical Center, Nashville, TN 37203, United States; Department of Health Policy, Vanderbilt University Medical Center, Nashville, TN 37203, United States; Veterans’ Health Administration Tennessee Valley Healthcare System, Geriatric Research Education and Clinical Center (GRECC), Nashville, TN 37212, United States; Department of Health Policy, Vanderbilt University Medical Center, Nashville, TN 37203, United States

**Keywords:** dose–response, observational study design, prescription drugs, simulation

## Abstract

Pharmacoepidemiologic studies commonly examine the association between drug dose and adverse health outcomes. In situations where no safe dose exists, the choice of modeling strategy can lead to identification of an apparent safe low dose range in the presence of a nonlinear relationship or due to the modeling strategy forcing a linear relationship through a dose of 0. We conducted a simulation study to assess the performance of several regression approaches to model the drug dose–response curve at low doses in a setting where no safe range exists, including the use of a (1) linear dose term, (2) categorical dose term, and (3) natural cubic spline terms. Additionally, we introduce and apply an expansion of prior work related to modeling dose–response curves at low and infrequently used doses in the setting of no safe dose (“spike-at-zero” and “slab-and-spline”). Furthermore, we demonstrate and empirically assess the use of these regression strategies in a practical scenario examining the association between the dose of the initial postpartum opioid prescribed after vaginal delivery and the subsequent total dose of opioids prescribed in the entire postpartum period among a cohort of opioid-naive women with a vaginal delivery enrolled in Tennessee’s Medicaid program (United States, 2007-2014).

## Introduction

Administrative data are regularly used to characterize associations between prescription drug doses and adverse treatment-related outcomes or events.^1^^‑^^3^ However, valid characterization of the full drug dose–response curve relies upon the appropriate choice of study design and modeling strategy to avoid the illusion of safe doses where none may truly exist. For example, linear modeling strategies can result in the misleading detection of a safe low-dose range in the presence of a nonlinear dose relationship, and categorical modeling strategies may be insufficiently powered to detect significant differences across dose categories.^4^^,^^5^ Flexible modeling approaches (eg, incorporation of cubic splines) are potential solutions to these issues, albeit ones that are more complex to implement and interpret. Yet even these approaches can be implemented in a way that presumes a “through-the-origin” relationship of the dose–response curve, inappropriately forcing confidence intervals for associations at low doses to approach and potentially include the null.^6^ Additionally, while the preferred active medication group comparisons can reduce confounding and bias concerns, few studies have examined opportunities to model dose–response associations in comparison to a zero-dose group (nonusers).^7^^,^^8^

One existing solution for modeling drug dose–response curves is the inclusion of a spike-at-zero term (ie, modeling the effect of an “infinitesimally small dose” for nonusers).^6^ This strategy serves as a straightforward adaptation of a continuous regression model that leverages the full range of data available and is compatible with flexible regression modeling approaches, although the utility and performance characteristics of this method for measuring associations are not yet well understood, particularly when observations in the low-dose range are infrequently observed.^6^ To that end, we conducted a simulation study to empirically assess the performance of the above-described regression approaches for modeling drug dose–response curves at low doses, with particular emphasis on the setting where no safe dose exists.

To illustrate their practical application, we further applied these regression approaches in a practical setting using administrative and pharmacy fill data to investigate the dose of prescribed opioids in the first four days postpartum as a predictor for subsequent doses of prescribed opioids during the remainder of the 42-day postpartum period. In the US, opioid prescribing remains incredibly common, even after uncomplicated vaginal delivery.^9^^,^^10^ As high-dose opioid prescriptions filled in the 42-day postpartum period have been linked to an increased subsequent risk of serious opioid-related harms (ie, persistent opioid use, opioid use disorder, opioid-related overdose), it is important to understand the association between the dosage of the first opioid prescription filled after delivery and the total dose of opioid prescriptions filled in the entire 42-day postpartum period.^11^^‑^^14^

## The “safe-dose” assumption and solutions

Let $X$ denote a nonnegative semi-continuous “dose” variable (with nonzero mass at $X=0$) and $Y$ a continuous outcome. For simplicity, our theoretical presentation does not involve covariate adjustment; when adjusting for covariates, all parameters characterized by the models possess corresponding conditional interpretations. Suppose we seek to estimate the dose–response function $\Delta (x)=\mathrm{E}\left[Y|X=x\right]-\mathrm{E}\left[Y|X=0\right]$ over a range of values for $x$. We define the safe-dose assumption as follows:

For any $\varepsilon >0$, there exists a $\delta >0$ such that $\left|\Delta (x)\right|<\mathrm{\varepsilon}$ for all $0<x<\mathrm{\delta}$.

In other words, the safe-dose assumption asserts that no matter how small a value we imagine for $\Delta (x)$—call that value $\mathrm{\varepsilon}$—there is a sufficiently small dose level (ie, $\mathrm{\delta}$ from the above definition) at which the effects of all lower doses (ie, $0<x<\mathrm{\delta} $) correspond to effects lying within that threshold (that is, $\left|\Delta (x)\right|$ must be within our initially imagined threshold, $\mathrm{\varepsilon}$). This assumption is embedded within many commonly implemented models, including the simple linear regression model $\mathrm{E}\left[Y|X=x\right]={\mathrm{\beta}}_0+{\mathrm{\beta}}_1x$. To see this, note that $\Delta (x)=x{\mathrm{\beta}}_1$, with $\underset{x\to{0}^{+}}{\lim}\Delta (x)=0$. More generally, the safe-dose assumption is assumed by any model taking the form $\mathrm{E}\left[Y|X=x\right]={\mathrm{\beta}}_0+{f}_{\boldsymbol{\mathrm{\beta}}}(x)$ such that $\underset{x\to{0}^{+}}{\lim }{f}_{\boldsymbol{\mathrm{\beta}}}(x)=0$, which includes most widely used basis spline models (eg, natural cubic splines).

**Figure 1:**
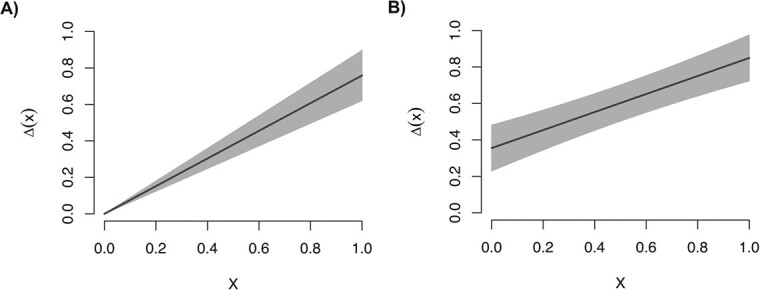
Simple illustration of the consequences associated with the safe-dose assumption. In this illustration, data are generated with a spike at zero. A) The dose–response curve as estimated by way of a simple linear regression model $\mathrm{E}\left[Y|X=x\right]={\mathrm{\beta}}_0+{\mathrm{\beta}}_1x$ (ie, with no spike); $\hat{\Delta}(x)$ is represented as a solid black line, with corresponding pointwise 95% confidence bands in gray. The estimated dose–response curve is forced through the origin, meaning that the value of $\Delta (0)$ is presumed to be zero, and the width of the confidence band continuously shrinks toward zero. B) In contrast, the estimated dose–response curve based on the model $\mathrm{E}\left[Y|X=x\right]={\beta}_0+{\beta}_11\left(x>0\right)+{\beta}_2x$ (ie, including a spike-at-zero term); the intercept of the dose–response curve is nonzero, and the confidence interval width does not shrink toward zero.

A corollary of the safe-dose assumption within a regression model is that its corresponding subgroup-specific standard errors continuously shrink toward zero with smaller doses. This can be readily seen, for instance, in a simple linear regression model, in which $\mathrm{Var}\big(\hat{\Delta}(x)\big)={x}^2\mathrm{Var}({\hat{\mathrm{\beta}}}_1)$. This is of practical importance as not only can one underrepresent the magnitude of $\Delta (x)$ at low doses, but one can simultaneously overstate confidence in that estimated effect (Figure 1A).

### The potential solution: inclusion of a “spike” effect

From a pharmacodynamic standpoint, the safe-dose assumption may very well be theoretically defensible: An infinitesimally small dose of a medication should theoretically produce nothing larger than an infinitesimally small effect. In the real world, however, filling a prescription puts someone on an inherently different trajectory, potentially creating outcome heterogeneity that is nonignorable even at small doses. This issue is separate from that of confounding by indication, in which differences in baseline characteristics between users and nonusers can produce bias. Confounding by indication is not the focus of this discussion, as even if proper techniques are employed to address confounding, the issue that produces a spike-at-zero would remain unaddressed. We seek to develop approaches that address this issue without excluding the nonusers from analysis.

One straightforward way to bypass the safe-dose assumption is to include a group-specific intercept for observations with a dose of zero (ie, a “spike-at-zero”).^6^ When modified in this way, the simple linear model takes the form $\mathrm{E}\left[Y|X=x\right]={\beta}_0+{\beta}_11\left(x>0\right)+{\beta}_2x$. In this case, we have that $\Delta (x)={\mathrm{\beta}}_1+{\mathrm{\beta}}_2x$. Here, $\underset{x\to{0}^{+}}{\lim}\Delta (x)={\mathrm{\beta}}_1$ theoretically represents the total effect of an infinitesimally small dose (henceforth referred to as the spike effect). Even for settings in which the spike effect is not a specific parameter of interest and/or cannot be reliably estimated, allowing a spike effect serves its primary purpose, which is to avoid the safe-dose assumption by acknowledging that exposure even to a very small dose would have a nonzero impact on the outcome; to see this, note that $\mathrm{Var}\big(\hat{\Delta}(x)\big)=\mathrm{Var}\big({\hat{\mathrm{\beta}}}_1\big)+{x}^2\mathrm{Var}\big({\hat{\mathrm{\beta}}}_2\big)+\mathrm{Cov}\big({\hat{\mathrm{\beta}}}_1,{\hat{\mathrm{\beta}}}_2\big)$, with $\underset{x\to 0}{\lim}\mathrm{Var}\big(\hat{\Delta}(x)\big)=\mathrm{Var}\big({\hat{\mathrm{\beta}}}_1\big)\ge 0$ (see Figure 1B).

It is often the case, however, that we wish to capture potential nonlinearity in the dose–response relationship. One simple way of doing so that implicitly avoids the safe-dose assumption is to discretize the continuous dose variable into categories, with a dose of zero included as one of the categories. This approach is understood to be limited by the unavoidably arbitrary specification of category boundaries and its failure to efficiently leverage available information across dose subgroups continuously. However, a spike effect can be incorporated into commonly implemented basis spline models (eg, natural cubic splines) with results analogous to those seen for a linear model which includes a spike.

### Slab effects for sparse regions

Continuous spline models serve to localize the use of degrees of freedom in a way so that uncertainty at each dose subgroup (eg, confidence interval width) is commensurate with the density (or sparsity) of locally available data. It is sometimes the case that low-dose subgroups are sparsely populated, in which case it may be appropriate to modify the left-most linear piece of a natural cubic spline model to instead be represented as a constant that joins the curve at the first knot in a continuously differentiable fashion (ie, impose a restriction of a zero slope to the left of the lower boundary knot). Although this is not a typically implemented basis expansion, it can be implemented with modifications to the natural cubic spline representation; we present the basis terms for this model and others discussed so far in Table S1, with graphical representations in Figure S1. We also provide supplementary methods and results in Appendix S1, and supplementary software code in Appendix S2 to generate a slab-and-spline basis expansion and extract point estimates and confidence intervals for both dose-specific means and dose-specific effects relative to zero using R (R Foundation for Statistical Computing, Vienna, Austria).

## Simulation study

### Simulation setup and methods

We conducted a simulation study to illustrate the utility of the spike-at-zero approaches when seeking to evaluate the association of low doses on a continuous outcome using various regression modeling approaches. We consider $M=5000$ simulation replicates under several scenarios, with the data generating mechanism described as follows. Let $N=1000$ denote the total sample size, each with a dose sampled from the following distribution:


\begin{align*} X\sim \left\{\begin{array}{@{}c}0\kern7.5em \mathrm{with}\ \mathrm{probability}\kern0.5em 0.25\\{}{\mathrm{\gamma}}_X\mathrm{Beta}\left({\mathrm{\alpha}}_X,5\right)\kern1.5em \mathrm{with}\ \mathrm{probability}\kern0.5em 0.75\end{array}\right.\! . \end{align*}

The parameters ${\alpha}_X$ and ${\gamma}_X$ control the distribution of nonzero doses (ie, with respect to both skewness and sparsity of data in the low-dose range). The following two data-generating mechanisms for $X$ are the primary focus of this simulation study. In the first (dose scenario 1), we set ${\mathrm{\alpha}}_X=1$, ${\mathrm{\beta}}_X=5$, and ${\mathrm{\gamma}}_X=2$, such that a higher proportion of observations are associated with low doses. In the second (dose scenario 2), we set ${\mathrm{\alpha}}_X=6$, ${\mathrm{\beta}}_X=5$, and ${\mathrm{\gamma}}_X=1$, such that a lower proportion of observations are associated with the low dose range (Figure S2).

The continuous outcome is generated under a quadratic model with a spike at zero: $Y=10+{\beta}_11\left(X>0\right)-2.4X+4.8{X}^2+\varepsilon$, where the independent errors are represented by $\mathrm{\varepsilon} \sim N\left(0,{\mathrm{\sigma}}_Y^2={1.5}^2\right)$. The parameter ${\mathrm{\beta}}_1$ marks the true spike effect to be estimated. Within each of dose scenario 1 and dose scenario 2, we consider two scenarios for the outcome: one in which there is a spike at zero (${\mathrm{\beta}}_1=0.5$) and one in which there is no spike (${\mathrm{\beta}}_1=0$).

In these examples, we compare the following regression models: (1) a simple linear regression model with no spike included; (2) a simple linear model with a spike at zero; (3) a quadratic model with a spike at zero—which, by the above-described data generating mechanism, is correctly specified; (4) a discretized-exposure model with dose categorized as 0, (0, 1/3], (1/3, 2/3], and >2/3; (5) a natural cubic spline model with knots at 1/8, 1/2, and 7/8 and no spike included; (6) a natural cubic spline with knots at 1/8, 1/2, and 7/8, and a spike at zero; (7) natural cubic spline with knots at 1/3, 1/2, and 2/3 and a spike at zero; and (8) a natural cubic spline with knots at 7/16, 5/8, and 13/16 and a slab over the interval [0, 7/16). We include an additional approach: (9) in which those in the zero-dose category are excluded from analysis; this approach instead compares each positive dose to the minimum observed dose group. In our secondary simulation scenarios, we compare the case in which there is no spike in the data-generating mechanism. Standard errors for point-wise effects were obtained from the Huber-White variance estimator. For all simulation settings, we estimate the average dose-response curve across various levels of $X$; we determine the average point-wise standard error across simulations, along with the corresponding empirical standard errors and coverage probabilities. All simulations were conducted using R, version 4.2.2.

### Simulation results: scenarios 1 and 2

**Figure 2:**
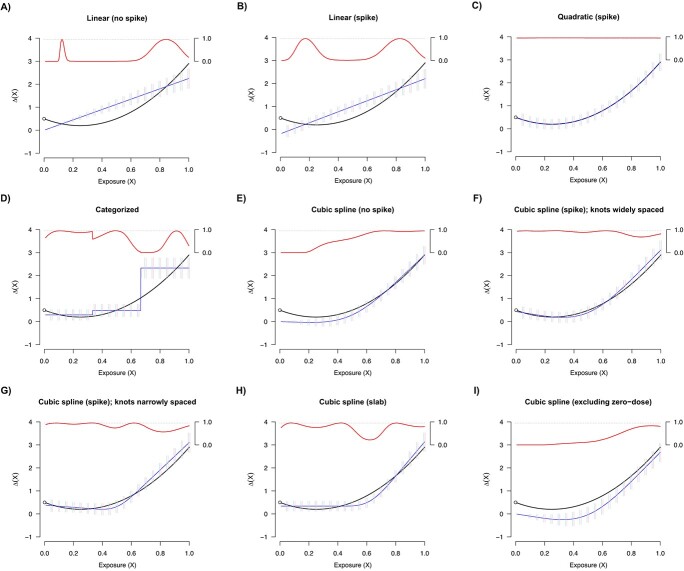
Simulation results in the presence of a spike effect for dose scenario 1 (data are dense in the lower dose range; see Figure S2). The true dose–response curve is shown as a solid black line. The average estimate across simulations is depicted as a solid blue line. Bars representing (1.96) times the empirical standard error (ie, the standard deviation of the estimates across simulations, shown in pink) and the average standard error (ie, the average of the estimated standard errors within dose groups across simulation, shown in light blue) are also shown at a variety of dose values. Further, the estimated coverage probabilities are presented as a function of dose as a solid red line, with a horizontal dash at 0.95 marking the target coverage threshold (secondary axis in the upper-right hand of each plot).

We first consider the setting in which there is a spike effect in the data-generating mechanism. The results corresponding to dose scenario 1 are shown in Figure 2. The numeric values for key operating characteristics of the simulations are shown in Table S2 at several dose levels. First, we note that across models, the average point-wise standard errors obtained from the model closely reflected the respective empirical standard errors over the range of doses. However, the behavior of standard error size as a function of dose varied substantially across models; for instance, the nonlinear models had standard errors that reflected the sparsity of data, and the standard errors from models relying on the safe-dose assumption continuously shrunk toward zero with lower doses.

The dose–response curve obtained from the quadratic model including a spike most closely reflected the truth, which was expected given that this parametric form was correctly specified. The models that do not include a spike serve as another illustration of the safe-dose assumption, as estimated curve is forced through the origin. The discretized model captures the nonlinearity, although very coarsely, as to be expected. On the other hand, the cubic spline models that feature a spike or a slab appear to be closely calibrated to the true dose–response curve, with coverage superior to that of the naive methods. The approach based on excluding zero-dose observations displays a downward shift, although the overall shape of the curve is similar to the other spline models (this model is estimating the effect relative to the minimum observed dose, a different target parameter than the one targeted by the other models).

**Figure 3:**
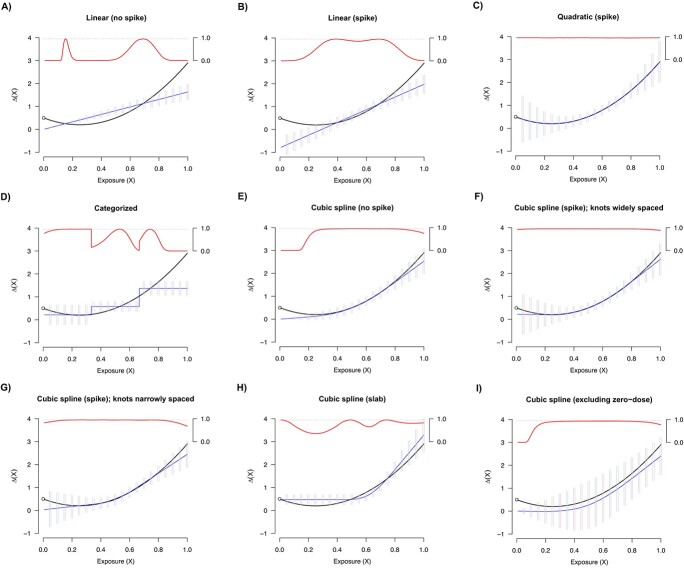
Simulation results in the presence of a spike effect for dose scenario 2 (data are sparse in the lower dose range; see Figure S2). The true dose–response curve is shown as a solid black line. The average estimate across simulations is depicted as a solid blue line. Bars representing (1.96) times the empirical standard error (ie, the standard deviation of the estimates across simulations, shown in pink) and the average standard error (ie, the average of the estimated standard errors within dose groups across simulation, shown in light blue) are also shown at a variety of dose values. Further, the estimated coverage probabilities are presented as a function of dose as a solid red line, with a horizontal dash at 0.95 marking the target coverage threshold (secondary axis in the upper-right hand of each plot).

The results corresponding to dose scenario 2 mirrored the results from dose scenario 1 (Figure 3; Table S2). Of note, the nonlinear models featuring a spike or a slab effect tended to have appropriately larger standard errors in the range of doses where data were more sparse, translating to superior coverage over other approaches.

For the secondary simulations in which the spike effect was zero, the results were largely analogous to the results described above, although the cubic spline model featuring no spike had proper performance (which is to be expected given that it accounts for nonlinearity; Figures S3 and S4; Table S3).

Across all simulations, this study illustrates the high degree of confidence in the low-dose range reflected by the models that invoke the safe-dose assumption.

## Practical application

### Practical application setup and methods

We compared the utility of the spike-at-zero and slab-based approaches with standard modeling approaches to examine the association between the initial discharge postpartum opioid dose and subsequent opioid use after delivery in the postpartum period among a retrospective cohort of patients who had a vaginal birth of a live infant from 2007-2014, and who were enrolled in the Tennessee Medicaid program.

**Figure 4:**
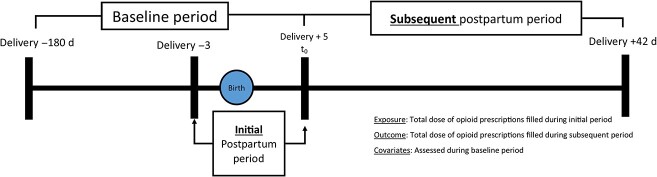
Characterization of baseline, cohort entry, exposure ascertainment window and outcome ascertainment window.

We examined the dose–response association between the initial opioid prescription filled within 4 days of delivery (initial postpartum period) and the subsequent total dose of opioid prescriptions filled in the subsequent postpartum period (day 5 through day 42 after delivery). Patients entered the cohort on postpartum day 5 after vaginal delivery (Figure 4). We included patients with evidence of a live birth, continuous enrollment during the baseline study period (180 days before delivery through day 4 after delivery), discharge on or before day 4 after delivery, and evidence of being opioid-naive during the baseline (≤ 1 outpatient opioid prescription). We excluded patients with evidence of an opioid use disorder or evidence of a filled methadone/buprenorphine prescription during the baseline period. We followed patients from day 5 after delivery through the earliest of loss of enrollment, death, or day 42 after delivery.

The primary exposure was the total morphine milligram equivalents (MME) of all opioid prescriptions filled during the initial postpartum period (defined as day 3 before delivery through day 4 after delivery, including boundary dates; see Figure 4). The primary outcome was the total MME of all opioid prescriptions filled during the subsequent postpartum period (defined as day 5 after delivery through day 42 after delivery, inclusive of boundary dates). We examined opioid prescriptions up to day 42 after delivery to reflect the standard 6-week postpartum period used in prior studies examining opioid prescribing in the postpartum period.^12^^,^^15^

**Figure 5:**
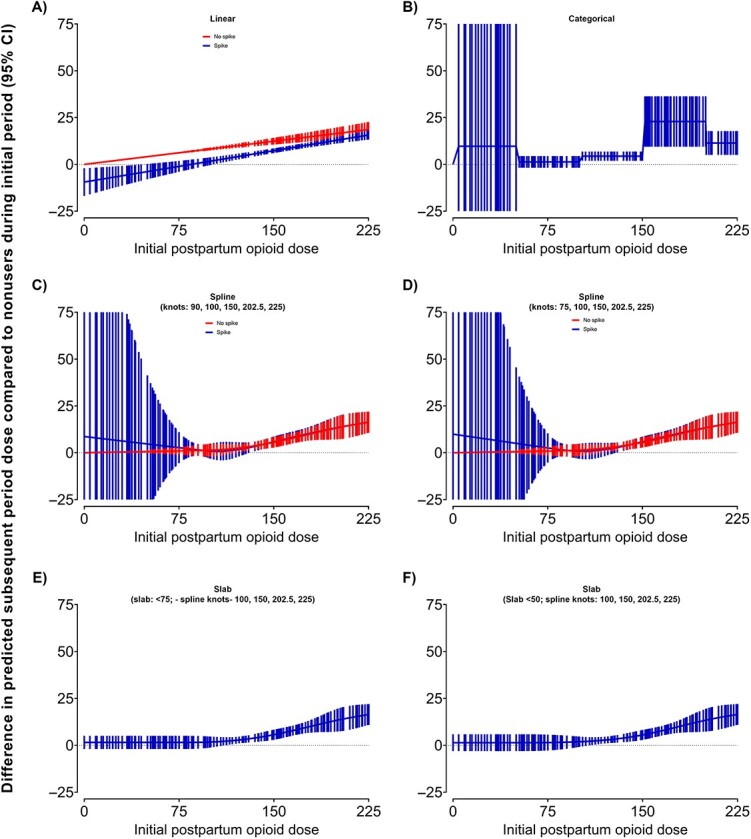
Difference in predicted dose in the subsequent postpartum period for each opioid dose observed in the initial period (y_x = i_) and the predicted dose in the subsequent postpartum period among those not filling an initial postpartum opioid prescription (y_x = 0_) (effect estimate = y_x = i_—y_x = 0_) in various modeling scenarios with and without a binary indicator term for any initial opioid prescription filled, adjusted for all relevant covariates and missing data with 50 imputations, Tennessee Medicaid (2007-2014). Note that effect estimates and confidence intervals were only calculated for observed dose values in the initial postpartum period; thus, gaps in the horizontal spacing of bars represent gaps in observed doses. Confidence intervals for portions of the *x*-axis extend outside the *y*-axis range (panels B, C, and D).

We explored 9 strategies to model the total opioid dose in the initial postpartum period in the regression, including linear models with and without a “spike” term, a categorical model based on 50-MME intervals, a natural cubic spline model with and without a “spike” term and different knot placements, and a “slab and spline” model with slab placements at different MME values (Figure 5).

In all scenarios and models, we adjusted for all covariates, used cluster-robust standard errors to account for patients with more than one delivery, and applied sequential multivariate imputation (50 iterations) to account for missing information for a small number of birth certificate variables (3.9%).^11^^,^^12^ Analyses were conducted using Stata, version 17.1 (StataCorp LP, College Station, TX). Further information about the data source, models, and formation of dose-specific confidence intervals can be found in the supplementary material (Appendix S1; Figure S5).

### Results

We identified 147 414 unique vaginal deliveries of a live infant among 113 702 unique mothers in the study population. Few patients experienced death or loss of enrollment up through day 42 after delivery (0.1%, *n* = 150). Most patients filled an opioid prescription during the initial postpartum period only (44.5%) or did not fill any opioid prescription during the full 42-day postpartum period (41.2%). A smaller number of patients filled an opioid prescription during both the initial and subsequent postpartum period (8.8%) or did not fill an opioid in the initial postpartum period but did fill an opioid prescription in the subsequent postpartum period (5.5%). Additional population characteristics and characteristics of the distribution of total opioid dose during the initial and subsequent postpartum period are highlighted in the supplementary material (Table S4 and Figure S6).

From each model type adjusting for all relevant covariates, we observed very similar dose response effect estimates and 95% confidence intervals for observed initial postpartum opioid doses > 225 MME (data not shown). However, we observed substantially different dose–response curves for total initial postpartum opioid doses < 225 MME, depending on the modeling strategy (Figure 5). All models without a “spike” term had a *y*-intercept of 0. Despite the small number of patients with an observed nonzero dose < 75 MME (*n* = 3624, 2.5%), the linear and spline models without a spike term produced dose–response curves with narrow confidence intervals for nonzero doses < 75 MME (Figure 5, red line in Figures  5A, 5C, and 5D). The linear model with a spike term predicted lower opioid doses in the subsequent postpartum period among those with an initial postpartum opioid dose < 75 MME compared to those without any initial postpartum opioid dose (Figure 5A, blue line). The spline models without a spike did not demonstrate any evidence that an initial dose < 75 MME led to an higher or lower subsequent opioid dose compared to those that did not fill an initial opioid (Figure 5, red line in Figures 5C and 5D). The categorical and spike and spline models did demonstrate a nonsignificantly elevated association at doses < 75 MME, albeit with very wide confidence intervals (Figure 5, blue lines in Figures 5B, 5C and 5D). The “slab-spline” approach demonstrated a nonsignificantly higher predicted subsequent opioid dose for initial doses < 100 MME like the spike and spline approach, although the method held the observed coefficients lower and with narrower confidence intervals (Figure 5, blue lines in Figures 5E and 5F).

## Discussion

The spike-and-spline and the slab-and-spline approaches provide advantages over traditional approaches in elucidating dose–response associations between continuous exposures and outcomes with markedly less bias, particularly at lower dose ranges. Given the relative ease of implementing a spike or slab effect, along with the very low cost in power, we recommend the inclusion of these terms to better capture the full dose–response relationship. In this sense, it is not necessary to specifically test for the presence of a spike effect in order to justify its use. Our simulation study illustrated a high degree of confidence in the low-dose range reflected by models invoking the safe-dose assumption; this is an assumption that is rewarded when it is correct but causes severe bias when it is incorrect. In our practical implementation of these novel approaches in the setting of the association between low-dose initial postpartum use and subsequent opioid use, we found that the interpretation of findings at low doses was sensitive to the modeling strategy in the presence of nonlinear relationships and infrequent low-dose prescriptions. Thus, studies of opioid doses should consider the use of flexible modeling approaches that do not presume a “through-the-origin” dose–response curve to prevent the illusion of a safe or effective dose where it does not exist. For practical purposes, the specific use of a spike term or a slab term is best guided by whether there are adequate data to support an additional degree of freedom in the low-dose range.

### Comparison to prior literature related to spike-at-zero and nonlinear dose–responses

Consideration for the impact of modeling strategy on the perceived dose–response of exposure-disease relationships has been explored outside of the pharmacoepidemiologic realm.^2^^,^^5^ For example, different modeling strategies for examining the association of low-dose environmental exposures on cancer risk have been previously described, including the importance of considering nonlinearity in the dose–response. A recent systematic review of cancer risk associated with low-dose, high-energy photon radiation reported 26 studies that specifically sought to examine how the choice of modeling strategy affects the interpretation of the risk of cancer associated with a radiation dose of 100 mGy or less.^5^ In short, the biological and epidemiologic evidence suggests that carcinogenicity at low doses is likely to be linear above doses > 10 mGy without any threshold effect, yet modeling strategies implemented for individual strategies can result in appropriately calculated confidence intervals that can affect the correct interpretation of the findings.^5^ Additionally, a small number of cases with high doses can have an influential impact on the modeled dose–response at lower dose ranges, especially if outcomes are rare at lower doses. However, the impact of the choice of modeling strategy on dose–response at lower prescription drug dose ranges in the setting of administrative databases is less well documented.

Assessments of drug dose–response using administrative data are often limited to comparisons of relative associations across categories of the drug dose exposure considering only a linear continuous term in the model.^1^^,^^16^^‑^^22^ And even recent work implementing flexible nonlinear modeling strategies can be limited by a “through-the-origin” modeling approach (ie, no “spike” or “slab” term) and by a lack of consideration for how the choice of modeling strategy affects the interpretation of findings at lower doses.^3^^,^^23^ Related work highlighted the importance of modeling exposure variables in populations with a high proportion of “unexposed” patients with a “spike-at-zero.”^6^ In simulation and real-world applied settings involving smoking and alcohol use, the authors have demonstrated the utility and improved measurement of the dose–response curves with the inclusion of a binary variable in the model for those with any nonzero dose exposure.^6^^,^^24^^,^^25^ The authors largely consider the incorporation of a “spike” effect in a fractional polynomial model (as opposed to the restricted cubic spline strategy outlined in our manuscript), and highlight several important considerations for the modeling of dose–response curves, including the difficulty in interpreting a risk function that does not start at 1 for a dose close to zero when implementing a “spike-at-zero” modeling approach. A major takeaway from their work is that the spike-at-zero approach is best applied to a research hypothesis focused on determining the best effect estimate for a common-exposure range, rather than trying to assess acceptable/effective lower limits of exposure. Our work expands this prior work to the pharmacoepidemiologic setting in which the exposure dose is measured using filled pharmacy claims data, which demonstrates the further benefit of modeling a spike effect to potential behavioral effects (nocebo or placebo) of filling a prescription and to more appropriately interpret confidence intervals for low dose values as compared to a “though-the-origin” dose–response curve approach.

### Limitations

The implementation and feasibility of the spike-and-spline and slab-and-spline flexible modeling approaches were only examined in the setting of a continuous exposure and outcome variable. Additional work is necessary to test the performance and applicability of these approaches in the setting of binary, categorical, and time-to-event outcomes. Further, it may be challenging in practice to fully prespecify all aspects of a model if the dose distribution is not well understood a priori. Dose distributions should be examined for possible sparsity in low-dose ranges to make a proper judgment on whether the leftmost linear piece of a natural cubic spline should be replaced by a slab.

The results of the practical application should be interpreted considering several potential limitations. We used filled pharmacy data to characterize opioid dose in the postpartum period.^26^^‑^^28^ Although allowing individuals to remain in the cohort even if they did not reach the end of the 42-day follow-up period could potentially introduce bias, excluding this very small number of individuals (*n* = 150, 0.1%) from analyses did not affect our estimates or conclusions. Although we accounted for an extensive list of potential confounders in the adjusted model using information from both claims, pharmacy, hospital discharge and birth certificate data, we cannot rule out the possibility of residual confounding due to missing covariates or misclassification of covariates. Of note, the inclusion of a spike or slab effect does not address issues surrounding confounding by indication, whereby treated individuals are likely systematically different from untreated individuals; alternative methods can help address such challenges. Finally, our findings may not be generalizable to privately insured women or women in other states.

### Conclusion

We recommend implementing a spike-and-spline or slab-and-spline approach in studies examining the dose–response association of prescription drug use with health outcomes when low-dose prescriptions are rare or unavailable.

## Supplementary Material

Web_Material_kwae147

## Data Availability

Sample code is available in our supplementary appendix material.
