# Supplementary material for: The impact of different strategies for modeling associations between medications at low doses and health outcomes: a simulation study and practical application to postpartum opioid use
Source: Am J Epidemiol. 2024 Jun 21;194(1):278–86. doi: 10.1093/aje/kwae147 (PMC11735964; doi:10.1093/aje/kwae147)

**The impact of different strategies for modeling associations between medications at low doses and health outcomes: a simulation study and practical application to postpartum opioid use**

Andrew J. Spieker, PhD; Margaret A. Adgent, PhD, MSPH; Sarah S. Osmundson, MD, MS; Sharon E. Phillips, MSPH; Ed Mitchel, Jr., MS; Ashley A. Leech, PhD, MS; Carlos G. Grijalva, MD, MPH; Andrew D. Wiese, MPH, PhD

**Supplementary Material**

**Contents:**

- **Appendix S1 –** Supplementary methods and results.
- **Appendix S2 –** Supplementary software code.
- **Table S1 –** Representation of several models and their presumed spike effects.
- **Table S2 –** Simulation results at key dose values in the presence of a spike effect.
- **Table S3 –** Simulation results at key dose values in the absence of a spike effect.
- **Table S4 –** Characteristics of patients based on opioid fill status across the 42-day postpartum period.
- **Figure S1 –** Graphical representation various models.
- **Figure S2 –** Characterization of dose distribution in each of two simulation scenarios.
- **Figure S3 –** Simulation results in the absence of a spike effect for dose scenario 1.
- **Figure S4 –** Simulation results in the absence of a spike effect for dose scenario 2.
- **Figure S5 –** Illustration with example code for slab effect.
- **Figure S6 –** Dose distribution during the initial and subsequent postpartum period.

**Appendix S1: Supplementary Methods and Results**

**METHODS**

**Data sources**

Data were derived from Tennessee Medicaid (TennCare) enrollment, claims, and pharmacy files; and supplemented with Vital Records data (birth and death certificates) and the Tennessee Hospital Discharge Data System (registry of all hospital-based encounters in Tennessee) to identify patient eligibility, healthcare encounters, and opioid prescriptions fills. The necessary institutional review boards approved the study.

**Covariates**

We identified baseline covariates (180 days before delivery through day 4 after delivery) including demographic information (e.g., age, race, sex), clinical conditions associated with pain and opioid use, contraindications to the use of non-steroid anti-inflammatory drugs, severe maternal morbidity, bilateral tubal ligation, perineal laceration, and mental health conditions.

**Computation of dose-specific 95% confidence intervals**

The effect estimate was calculated as the difference in predicted dose in the subsequent postpartum period for each opioid dose observed in the initial period (y_x=i_) and the predicted dose in the subsequent postpartum period among those not filling an initial postpartum opioid (y_x=0_) [effect estimate = y_x=i_ – y_x=0_]. As an example, let us consider the setting in which the predicted dose of opioids filled in the subsequent postpartum period was 50 MME for those with an initial postpartum opioid dose of 100 MME (y_x=100_ = 50) and 25 MME for those with an initial postpartum opioid dose of 0 (i.e., no opioid filled in the initial period) [y_x=0_ = 25). In this example, the point estimate for the effect at an initial opioid dose of 100 MME would 25 MME (effect estimate = y_x=100_ – y_x=0_ = 50 – 25).

**RESULTS**

**Distribution of opioid dose**

The median total dose was highest among those that filled an opioid during both postpartum periods (300 MME; IQR: 225-450) compared to those that filled an opioid only during the initial postpartum opioid (150 MME; IQR: 100-200) and only during the subsequent postpartum period (142.5 MME; 100-225). Though the medians were similar (150 vs. 142.5 MME), the mean opioid dose in the subsequent postpartum period was higher among those that also filled an opioid in the initial postpartum period (mean: 237.1 MME) compared to those that did fill an opioid in the initial period (mean: 198.5 MME). Also see **Figure S6**.

**Appendix S2: Supplementary Software Code**

*The purpose of this supplementary software code is to provide a function with the capability of performing the slab-and-spline basis expansion on a single numeric variable in R. These functions presume that the data loaded into the function are complete; these functions are not designed to find and troubleshoot errors.*

## This function performs the basis expansion for a natural cubic spline that transforms

## the left-most linear piece into a constant

## **x** : continuous numeric variable on which to perform the basis expansion

## **knots** : specific knot positions (minimum: 3; defaults to quantiles of nonzero values)

## **stub** : denotes the “prefix” with which to label the resulting columns

slab.spline <- function(x, knots = NULL, stub = "n") {

if (is.null(knots)) {knots <- quantile(x[x > 0], c(0.2, 0.5, 0.8))}

N <- length(x)

P <- length(knots)

xp <- as.numeric(x > 0)

xsl <- as.numeric(x > knots[1])

zP <- knots[P]

zP.1 <- knots[P - 1]

bmat <- matrix(0, nrow = N, ncol = P - 1)

bmat[,1] <- xp

nms <- c(paste(stub, 1, sep = ""))

for (j in 1:(P - 2)) {

zp <- knots[j]

dp.num <- pmax(0, (x - zp)^3) - pmax(0, (x - zP)^3)

dp <- dp.num/(zP - zp)

dP.1.num <- pmax(0, (x - zP.1)^3) - pmax(0, (x - zP)^3)

dP.1 <- dP.1.num/(zP - zP.1)

bmat[,j + 1] <- (dp - dP.1)*xsl

nms <- c(nms, paste(stub, j + 1, sep = ""))

}

bmat <- data.frame(bmat)

names(bmat) <- nms

return(bmat)

}

## This function extracts point estimates and confidence intervals for either the subgroup-

## specific mean of the outcome or the estimated dose effect relative to a zero-dose

## **model** : continuous numeric variable on which to perform the basis expansion

## **new.x** : specific knot positions (minimum: 3; defaults to quantiles of nonzero values)

## **knots** : specification must be compatible with slab.spline()

## **stub** : specification must be compatible with slab.spline()

## **alpha** : desired confidence level (defaults to 0.05)

## **type** : specification of whether mean or effect estimates are preferred

predict.slab.model <- function(model, new.x, knots, stub = "n", alpha = 0.05, type = "mean") {

if (type == "mean") {int <- 1}

if (type == "difference") {int <- 0}

new.slab.expansion <- slab.spline(x = new.x, knots = knots, stub = stub)

pr <- predict(model, newdata = new.slab.expansion, se.fit = TRUE)

pr.val <- pr$fit

pr.ses <- pr$se.fit

R <- matrix(rep(int*coef(model)[1], length(pr.val)), ncol = 1)

for (j in 2:(length(coef(model)))) {

R <- cbind(R, new.slab.expansion[,j - 1]*coef(model)[j])

}

S <- matrix(rep(int*coef(model)[1], length(pr.val)), ncol = 1)

for (j in 2:(length(coef(model)))) {

S <- cbind(S, new.slab.expansion[,j - 1])

}

EST.DIFF <- R %*% matrix(c(int, rep(1, length(coef(model)) - 1)), ncol = 1)

SE.DIFF <- sqrt(diag(S %*% vcov(model) %*% t(S)))

out <- data.frame(cbind(new.x, EST.DIFF,

EST.DIFF - qnorm(1 - alpha/2)*SE.DIFF,

EST.DIFF + qnorm(1 - alpha/2)*SE.DIFF))

names(out) <- c("X", "EST", "CILOW", "CIHI")

return(out)

}

## Set working directory first

## Read in the data file

dat <- read.csv("slab-example.csv")

## Perform the slab-expansion on the exposure

slab.expansion <- slab.spline(dat$X, knots = c(0.2, 0.3, 0.9), stub = "x.slab")

## Append the basis expansion to the data set

dat <- cbind(dat, slab.expansion)

## Preview of the result

## > head(dat)

## ID X Y x.slab1 x.slab2

## 1 1 0.57 7.64 1 0.03955643

## 2 2 0.18 5.84 1 0.00000000

## 3 3 0.82 7.82 1 0.10612190

## 4 4 0.44 5.51 1 0.01517524

## 5 5 0.62 6.25 1 0.05122667

## 6 6 0.04 4.92 1 0.00000000

## Fit the model

model <- lm(Y ~ x.slab1 + x.slab2, data = dat)

## Create a scatter plot of the data

plot(dat$X, dat$Y, xlim = c(0,1), ylim = c(2, 10), frame.plot = FALSE,

xlab = "X", ylab = "Y", pch = 20, cex = 0.8, col = "gray80",

main = "(A) \n Dose-specific mean estimates")

## Generate and plot estimates and 95% confidence intervals for the dose-specific means

pred.mean <- predict.slab.model(model = model, new.x = seq(0.001, 1, 0.001),

knots = c(0.2, 0.3, 0.9), stub = "x.slab", alpha = 0.05)

lines(pred.mean$X, pred.mean$EST, col = "red", lwd = 2)

lines(pred.mean$X, pred.mean$CILOW, col = "pink", lwd = 2)

lines(pred.mean$X, pred.mean$CIHI, col = "pink", lwd = 2)

## Generate and plot estimates and 95% confidence intervals for dose-specific differences

## (meaning, relative to zero-dose)

plot(c(0,0), col = "white",

xlim = c(0,1), ylim = c(0, 2.5),

frame.plot = FALSE,

xlab = "X", ylab = expression(paste(Delta(X))),

main = "(B) \n Differences relative to zero-dose")

pred.diff <- predict.slab.model(model = model, new.x = seq(0.001, 1, 0.001),

knots = c(0.2, 0.3, 0.9), stub = "x.slab", alpha = 0.05,

type = "difference")

lines(pred.diff$X, pred.diff$EST, col = "blue", lwd = 2)

lines(pred.diff$X, pred.diff$CILOW, col = "lightblue", lwd = 2)

lines(pred.diff$X, pred.diff$CIHI, col = "lightblue", lwd = 2)

**Table S1**. Representation of several models and their presumed spike effects. Note that $h_{k}\left( x;\boldsymbol{\zeta} \right)$ denotes the $k$^th^ basis function for a natural cubic spline with knots at $\zeta_{1}$, …, $\zeta_{K}$, for $k=1,\ldots,K$.

| Model description | Model: $E\left[ Y \vert X=x \right]$ | $\lim_{x\to0^{+}} \Delta\left( x \right)$ | Interpretation |
| --- | --- | --- | --- |
| Simple linear (no spike) | $\beta_{0}+\beta_{1}x$ | 0 | Invokes the safe-dose assumption. |
| Simple linear (spike) | $\beta_{0}+\beta_{1}1\left( x>0 \right)+\beta_{3}x$ | $\beta_{1}$ | Allows effect of an infinitesimally small dose. |
| Categorized (spike) | $\beta_{0}+\beta_{1}1\left( x>0 \right)+\sum_{k=1}^{K-2} \beta_{k+1}1\left( \zeta_{k+1}<x\leq\zeta_{k+2} \right)+\beta_{K}1\left( x>\zeta_{K} \right)$ | $\beta_{1}$ | Models average effect over $\left( 0, \zeta_{1} \right)$. |
| Natural cubic spline (no spike) | $\beta_{0}+\sum_{k=1}^{K} \beta_{k}h_{k+1}\left( x; \boldsymbol{\zeta} \right)$ | 0 | Safe-dose assumption invoked. |
| Natural cubic spline (spike) | $\beta_{0}+\beta_{1}1\left( x>0 \right)+\sum_{k=1}^{K-1} \beta_{k+1}h_{k+1}\left( x; \boldsymbol{\zeta} \right)$ | $\beta_{1}$ | Allows effect of an infinitesimally small dose. |
| Natural cubic spline (slab) | $\beta_{0}+\beta_{1}1\left( x>0 \right)+\sum_{k=1}^{K-2} \beta_{k+1}h_{k+2}\left( x; \boldsymbol{\zeta} \right)1\left( x>\zeta_{1} \right)$ | $\beta_{1}$ | Models average effect over $\left( 0, \zeta_{1} \right)$. |

**Table S2**. Simulation results at key dose values in the presence of a spike effect. In dose scenario 1, data are dense in the lower dose range, whereas in dose scenario 2, the data are sparse in the lower dose range (see **Figure S2**). Depicted are the average estimates (est.), empirical standard error across simulations (ESE), average estimated standard error (ASE), and coverage probability (CP) for each of the nine methods at key values of the dose.

|  | **X = 0.00 (Δ = 0.50)** | | | | **X = 0.25 (Δ = 0.20)** | | | | **X = 0.50 (Δ = 0.50)** | | | | **X = 0.75 (Δ = 1.40)** | | | | **X = 1.00 (Δ = 2.90)** | | | |
| --- | --- | --- | --- | --- | --- | --- | --- | --- | --- | --- | --- | --- | --- | --- | --- | --- | --- | --- | --- | --- |
| Dose scenario 1 | Est. | ESE | ASE | CP | Est. | ESE | ASE | CP | Est. | ESE | ASE | CP | Est. | ESE | ASE | CP | Est. | ESE | ASE | CP |
| [1] Linear | 0.00 | 0.000 | 0.000 | 0.00 | 0.56 | 0.055 | 0.055 | 0.00 | 1.12 | 0.110 | 0.110 | 0.00 | 1.69 | 0.165 | 0.165 | 0.58 | 2.25 | 0.219 | 0.220 | 0.17 |
| [2] Linear + spike | -0.19 | 0.136 | 0.136 | 0.00 | 0.41 | 0.112 | 0.112 | 0.52 | 1.01 | 0.122 | 0.122 | 0.01 | 1.61 | 0.161 | 0.161 | 0.74 | 2.21 | 0.212 | 0.213 | 0.10 |
| [3] Quadratic (spike) | 0.50 | 0.147 | 0.148 | 0.95 | 0.20 | 0.113 | 0.113 | 0.95 | 0.50 | 0.127 | 0.127 | 0.95 | 1.40 | 0.139 | 0.139 | 0.95 | 2.90 | 0.188 | 0.189 | 0.95 |
| [4] Categorized | 0.00 | 0.000 | 0.000 | 0.00 | 0.29 | 0.119 | 0.119 | 0.88 | 0.49 | 0.141 | 0.143 | 0.95 | 2.33 | 0.229 | 0.227 | 0.01 | 2.33 | 0.229 | 0.227 | 0.30 |
| [5] Spline (no spike) | 0.00 | 0.000 | 0.000 | 0.00 | -0.03 | 0.082 | 0.083 | 0.20 | 0.29 | 0.128 | 0.130 | 0.63 | 1.42 | 0.132 | 0.133 | 0.95 | 2.89 | 0.200 | 0.200 | 0.95 |
| [6] Spline + spike (wide) | 0.44 | 0.146 | 0.146 | 0.93 | 0.21 | 0.113 | 0.114 | 0.95 | 0.40 | 0.133 | 0.134 | 0.89 | 1.55 | 0.139 | 0.139 | 0.81 | 3.11 | 0.212 | 0.212 | 0.82 |
| [7] Spline + spike (narrow) | 0.38 | 0.143 | 0.143 | 0.87 | 0.26 | 0.112 | 0.113 | 0.92 | 0.32 | 0.139 | 0.140 | 0.74 | 1.62 | 0.140 | 0.140 | 0.64 | 3.10 | 0.215 | 0.215 | 0.83 |
| [8] Spline + slab | 0.34 | 0.111 | 0.111 | 0.69 | 0.34 | 0.111 | 0.111 | 0.76 | 0.35 | 0.111 | 0.111 | 0.72 | 1.28 | 0.120 | 0.120 | 0.82 | 3.13 | 0.207 | 0.208 | 0.79 |
| [9] Exclude non-users | -0.98 | 0.428 | 0.432 | 0.07 | -0.23 | 0.105 | 0.106 | 0.02 | -0.03 | 0.169 | 0.170 | 0.12 | 1.11 | 0.165 | 0.166 | 0.57 | 2.68 | 0.207 | 0.208 | 0.81 |
| Dose scenario 2 | Est. | ESE | ASE | CP | Est. | ESE | ASE | CP | Est. | ESE | ASE | CP | Est. | ESE | ASE | CP | Est. | ESE | ASE | CP |
| [1] Linear | 0.00 | 0.000 | 0.000 | 0.00 | 0.41 | 0.045 | 0.045 | 0.00 | 0.82 | 0.090 | 0.090 | 0.06 | 1.22 | 0.136 | 0.135 | 0.74 | 1.63 | 0.181 | 0.180 | 0.00 |
| [2] Linear + spike | -0.79 | 0.238 | 0.238 | 0.00 | -0.10 | 0.158 | 0.159 | 0.52 | 0.59 | 0.111 | 0.111 | 0.87 | 1.28 | 0.136 | 0.136 | 0.86 | 1.98 | 0.209 | 0.208 | 0.01 |
| [3] Quadratic (spike) | 0.50 | 0.617 | 0.624 | 0.95 | 0.20 | 0.205 | 0.208 | 0.95 | 0.50 | 0.119 | 0.119 | 0.95 | 1.40 | 0.145 | 0.145 | 0.95 | 2.90 | 0.456 | 0.460 | 0.95 |
| [4] Categorized | 0.00 | 0.000 | 0.000 | 0.00 | 0.21 | 0.217 | 0.222 | 0.95 | 0.58 | 0.116 | 0.116 | 0.90 | 1.36 | 0.155 | 0.154 | 0.94 | 1.36 | 0.155 | 0.154 | 0.00 |
| [5] Spline (no spike) | 0.00 | 0.000 | 0.000 | 0.00 | 0.13 | 0.083 | 0.083 | 0.86 | 0.49 | 0.120 | 0.120 | 0.95 | 1.39 | 0.141 | 0.140 | 0.95 | 2.53 | 0.288 | 0.286 | 0.75 |
| [6] Spline + spike (wide) | 0.22 | 0.504 | 0.510 | 0.92 | 0.21 | 0.208 | 0.210 | 0.95 | 0.49 | 0.121 | 0.120 | 0.95 | 1.41 | 0.146 | 0.146 | 0.95 | 2.62 | 0.351 | 0.353 | 0.88 |
| [7] Spline + spike (narrow) | 0.03 | 0.438 | 0.444 | 0.81 | 0.20 | 0.208 | 0.210 | 0.95 | 0.47 | 0.124 | 0.124 | 0.95 | 1.41 | 0.147 | 0.147 | 0.95 | 2.45 | 0.296 | 0.298 | 0.68 |
| [8] Spline + slab | 0.47 | 0.115 | 0.115 | 0.94 | 0.47 | 0.115 | 0.115 | 0.36 | 0.48 | 0.115 | 0.115 | 0.95 | 1.42 | 0.148 | 0.148 | 0.95 | 3.28 | 0.378 | 0.380 | 0.82 |
| [9] Exclude non-users | -0.08 | 1.315 | 1.328 | 0.93 | -0.01 | 0.324 | 0.327 | 0.90 | 0.27 | 0.528 | 0.533 | 0.93 | 1.19 | 0.486 | 0.489 | 0.92 | 2.40 | 0.418 | 0.418 | 0.77 |

**Table S3**. Simulation results at key dose values in the absence of a spike effect. In dose scenario 1, data are dense in the lower dose range, whereas in dose scenario 2, the data are sparse in the lower dose range (see **Figure S2**). Depicted are the average estimates (est.), empirical standard error across simulations (ESE), average estimated standard error (ASE), and coverage probability (CP) for each of the nine methods at key values of the dose.

|  | **X = 0.00 (Δ = 0.50)** | | | | **X = 0.25 (Δ = 0.20)** | | | | **X = 0.50 (Δ = 0.50)** | | | | **X = 0.75 (Δ = 1.40)** | | | | **X = 1.00 (Δ = 2.90)** | | | |
| --- | --- | --- | --- | --- | --- | --- | --- | --- | --- | --- | --- | --- | --- | --- | --- | --- | --- | --- | --- | --- |
| Dose scenario 1 | Est. | ESE | ASE | CP | Est. | ESE | ASE | CP | Est. | ESE | ASE | CP | Est. | ESE | ASE | CP | Est. | ESE | ASE | CP |
| [1] Linear | 0.00 | 0.000 | 0.000 | 1.00 | 0.46 | 0.057 | 0.057 | 0.00 | 0.93 | 0.113 | 0.115 | 0.00 | 1.39 | 0.170 | 0.172 | 0.18 | 1.86 | 0.227 | 0.230 | 0.35 |
| [2] Linear + spike | -0.68 | 0.137 | 0.136 | 0.00 | -0.09 | 0.112 | 0.112 | 0.52 | 0.51 | 0.122 | 0.122 | 0.01 | 1.11 | 0.160 | 0.161 | 0.75 | 1.71 | 0.211 | 0.213 | 0.10 |
| [3] Quadratic (spike) | 0.00 | 0.149 | 0.148 | 0.95 | -0.30 | 0.114 | 0.113 | 0.95 | 0.00 | 0.127 | 0.127 | 0.95 | 0.90 | 0.138 | 0.139 | 0.95 | 2.40 | 0.190 | 0.189 | 0.95 |
| [4] Categorized | 0.00 | 0.000 | 0.000 | 1.00 | -0.21 | 0.119 | 0.119 | 0.87 | -0.01 | 0.140 | 0.143 | 0.95 | 1.83 | 0.227 | 0.227 | 0.02 | 1.83 | 0.227 | 0.227 | 0.29 |
| [5] Spline (no spike) | 0.00 | 0.000 | 0.000 | 1.00 | -0.26 | 0.081 | 0.082 | 0.92 | -0.08 | 0.127 | 0.129 | 0.91 | 1.07 | 0.131 | 0.132 | 0.76 | 2.64 | 0.198 | 0.197 | 0.76 |
| [6] Spline + spike (wide) | -0.06 | 0.147 | 0.146 | 0.93 | -0.29 | 0.114 | 0.114 | 0.95 | -0.10 | 0.132 | 0.134 | 0.89 | 1.05 | 0.139 | 0.139 | 0.82 | 2.61 | 0.214 | 0.212 | 0.82 |
| [7] Spline + spike (narrow) | -0.12 | 0.145 | 0.143 | 0.87 | -0.24 | 0.113 | 0.113 | 0.91 | -0.18 | 0.138 | 0.140 | 0.76 | 1.12 | 0.140 | 0.140 | 0.64 | 2.60 | 0.217 | 0.216 | 0.83 |
| [8] Spline + slab | -0.16 | 0.112 | 0.111 | 0.69 | -0.16 | 0.112 | 0.111 | 0.75 | -0.15 | 0.111 | 0.111 | 0.72 | 0.78 | 0.121 | 0.120 | 0.83 | 2.63 | 0.211 | 0.208 | 0.79 |
| [9] Exclude non-users | -0.98 | 0.431 | 0.432 | 0.38 | -0.23 | 0.106 | 0.106 | 0.89 | -0.03 | 0.170 | 0.170 | 0.95 | 1.11 | 0.166 | 0.166 | 0.76 | 2.67 | 0.209 | 0.209 | 0.74 |
| Dose scenario 2 | Est. | ESE | ASE | CP | Est. | ESE | ASE | CP | Est. | ESE | ASE | CP | Est. | ESE | ASE | CP | Est. | ESE | ASE | CP |
| [1] Linear | 0.00 | 0.000 | 0.000 | 1.00 | 0.23 | 0.045 | 0.046 | 0.00 | 0.46 | 0.090 | 0.091 | 0.00 | 0.69 | 0.135 | 0.137 | 0.65 | 0.91 | 0.181 | 0.182 | 0.00 |
| [2] Linear + spike | -1.29 | 0.240 | 0.238 | 0.00 | -0.60 | 0.159 | 0.159 | 0.53 | 0.09 | 0.110 | 0.111 | 0.87 | 0.78 | 0.135 | 0.135 | 0.86 | 1.47 | 0.208 | 0.207 | 0.01 |
| [3] Quadratic (spike) | 0.00 | 0.612 | 0.622 | 0.95 | -0.30 | 0.204 | 0.207 | 0.95 | 0.00 | 0.118 | 0.118 | 0.95 | 0.90 | 0.145 | 0.145 | 0.95 | 2.40 | 0.460 | 0.459 | 0.95 |
| [4] Categorized | 0.00 | 0.000 | 0.000 | 1.00 | -0.28 | 0.218 | 0.222 | 0.95 | 0.08 | 0.115 | 0.116 | 0.90 | 0.86 | 0.154 | 0.154 | 0.94 | 0.86 | 0.154 | 0.154 | 0.00 |
| [5] Spline (no spike) | 0.00 | 0.000 | 0.000 | 1.00 | -0.18 | 0.083 | 0.083 | 0.70 | -0.02 | 0.119 | 0.120 | 0.95 | 0.93 | 0.140 | 0.140 | 0.95 | 2.23 | 0.290 | 0.286 | 0.90 |
| [6] Spline + spike (wide) | -0.28 | 0.500 | 0.508 | 0.91 | -0.29 | 0.207 | 0.210 | 0.95 | -0.01 | 0.119 | 0.120 | 0.95 | 0.91 | 0.146 | 0.146 | 0.95 | 2.11 | 0.355 | 0.352 | 0.87 |
| [7] Spline + spike (narrow) | -0.47 | 0.434 | 0.443 | 0.81 | -0.30 | 0.207 | 0.210 | 0.95 | -0.03 | 0.123 | 0.124 | 0.94 | 0.91 | 0.147 | 0.147 | 0.95 | 1.94 | 0.300 | 0.297 | 0.66 |
| [8] Spline + slab | -0.03 | 0.114 | 0.115 | 0.94 | -0.03 | 0.114 | 0.115 | 0.34 | -0.02 | 0.114 | 0.115 | 0.95 | 0.91 | 0.148 | 0.148 | 0.95 | 2.77 | 0.383 | 0.379 | 0.83 |
| [9] Exclude non-users | -0.07 | 1.302 | 1.324 | 0.95 | -0.01 | 0.321 | 0.326 | 0.85 | 0.27 | 0.522 | 0.532 | 0.92 | 1.19 | 0.479 | 0.487 | 0.91 | 2.40 | 0.414 | 0.417 | 0.95 |

**Table S4.** Characteristics of patients based on opioid fill status across the 42-day postpartum period.

| Characteristic | Total | No opioid | Opioid fill only during initial postpartum period | Opioid fill only during subsequent postpartum period | Opioid fill in both periods |
| --- | --- | --- | --- | --- | --- |
| N | 147,414 | 60,731 (41.2%) | 65,612 (44.5%) | 8,131 (5.5%) | 12,940 (8.8%) |
| Mean age (sd) | 23.6 (5.0) | 23.6 (5.2) | 23.4 (4.9) | 23.7 (5.2) | 23.7 (4.9) |
| Median age (IQR) | 23.0 (20.0-26.0) | 23 (16-30) | 22 (16-28) | 23 (16-30) | 23 (16-30) |
|  |  |  |  |  |  |
| Mean distance to hospital (sd) | 11.8 (12.3) | 11.4 (12.6) | 12.1 (12.1) | 12.1 (12.3) | 12.1 (12.1) |
| Median distance to hospital | 8.0 (3.8-16.0) | 7.4 | 8.5 | 8.2 | 8.7 |
|  |  |  |  |  |  |
| Race/ethnicity |  |  |  |  |  |
| *White* | 89,551 (61%) | 30,774 (51%) | 44,116 (67%) | 4,639 (57%) | 10,022 (77%) |
| *Non-white* | 57,774 (39%) | 29,907 (49%) | 21,466 (33%) | 3,488 (43%) | 2,913 (23%) |
| Missing | 89 (0%) | 50 (0%) | 30 (0%) | 4 (0%) | 5 (0%) |
|  |  |  |  |  |  |
| Parity |  |  |  |  |  |
| *0* | 59,759 (41%) | 23,482 (39%) | 28,193 (43%) | 3,084 (38%) | 5,000 (39%) |
| *1* | 43,437 (29%) | 17,842 (29%) | 19,423 (30%) | 2,290 (28%) | 3,882 (30%) |
| *>=2* | 43,757 (30%) | 19,265 (32%) | 17,748 (27%) | 2,739 (34%) | 4,005 (31%) |
| Missing | 461 (0%) | 142 (0%) | 248 (0%) | 18 (0%) | 53 (0%) |
|  |  |  |  |  |  |
| Plurality |  |  |  |  |  |
| *1* | 146,562 (99%) | 60,390 (99%) | 65,243 (99%) | 8,071 (99%) | 12,858 (99%) |
| *2 or more* | 852 (0%) | 341 (0%) | 369 (0%) | 60 (1%) | 82 (1%) |
|  |  |  |  |  |  |
| Income bracket |  |  |  |  |  |
| *$0-34,999* | 30,459 (21%) | 15,024 (25%) | 11,427 (17%) | 1,839 (23%) | 2,169 (17%) |
| *$35,000-44,999* | 36,806 (25%) | 15,435 (25%) | 15,961 (24%) | 2,096 (26%) | 3,314 (26%) |
| *$45,000-59,999* | 34,658 (24%) | 13,150 (22%) | 16,358 (25%) | 1,905 (23%) | 3,245 (25%) |
| *>=$60,000* | 40,218 (27%) | 14,507 (24%) | 19,933 (30%) | 1,947 (24%) | 3,831 (30%) |
| Missing | 5,273 (4%) | 2,615 (4%) | 1,933 (3%) | 344 (4%) | 381 (3%) |
|  |  |  |  |  |  |
| Region |  |  |  |  |  |
| *West* | 49,676 (34%) | 28,942 (48%) | 15,107 (23%) | 3,082 (38%) | 2,545 (20%) |
| *Central* | 49,305 (33%) | 15,108 (25%) | 26,563 (40%) | 2,435 (30%) | 5,199 (40%) |
| *East* | 48,005 (33%) | 16,399 (27%) | 23,831 (36%) | 2,598 (32%) | 5,177 (40%) |
| Missing | 428 (0%) | 282 (0%) | 111 (0%) | 16 (0%) | 19 (0%) |
|  |  |  |  |  |  |
| Hospitalization days |  |  |  |  |  |
| *0* | 478 (0%) | 214 (0%) | 206 (0%) | 23 (0%) | 35 (0%) |
| *1* | 41,637 (28%) | 19,742 (33%) | 16,811 (26%) | 1,939 (24%) | 3,145 (24%) |
| *2* | 99,683 (68%) | 38,761 (64%) | 46,090 (70%) | 5,727 (70%) | 9,105 (70%) |
| *3* | 4,958 (3%) | 1,775 (3%) | 2,262 (3%) | 353 (4%) | 568 (4%) |
| *4* | 658 (0%) | 239 (0%) | 243 (0%) | 89 (1%) | 87 (1%) |
|  |  |  |  |  |  |
| Pre-delivery hospitalizations |  |  |  |  |  |
| *0 or 1* | 91,221 (62%) | 40,459 (67%) | 39,105 (60%) | 4,664 (57%) | 6,993 (54%) |
| *2* | 29,669 (20%) | 11,347 (19%) | 13,805 (21%) | 1,710 (21%) | 2,807 (22%) |
| *3 or more* | 26,524 (18%) | 8,925 (15%) | 12,702 (19%) | 1,757 (22%) | 3,140 (24%) |
|  |  |  |  |  |  |
| Pre-delivery ED visits |  |  |  |  |  |
| *0* | 84,377 (57%) | 34,592 (57%) | 38,762 (59%) | 4,305 (53%) | 6,718 (52%) |
| *1* | 36,319 (25%) | 14,615 (24%) | 16,177 (25%) | 2,113 (26%) | 3,414 (26%) |
| *2 or more* | 26,718 (18%) | 11,524 (19%) | 10,673 (16%) | 1,713 (21%) | 2,808 (22%) |
|  |  |  |  |  |  |
| Pre-delivery outpatient |  |  |  |  |  |
| *0* | 39,117 (27%) | 17,980 (30%) | 16,348 (25%) | 1,826 (22%) | 2,963 (23%) |
| *1 to 3* | 76,124 (52%) | 30,711 (51%) | 34,470 (53%) | 4,162 (51%) | 6,781 (52%) |
| *4 or more* | 32,173 (22%) | 12,040 (20%) | 14,794 (23%) | 2,143 (26%) | 3,196 (25%) |
|  |  |  |  |  |  |
| Year of delivery |  |  |  |  |  |
| *2007* | 19,256 (13%) | 8,185 (13%) | 8,201 (12%) | 1,108 (14%) | 1,762 (14%) |
| *2008* | 19,315 (13%) | 7,485 (12%) | 8,717 (13%) | 1,139 (14%) | 1,974 (15%) |
| *2009* | 19,303 (13%) | 7,249 (12%) | 9,024 (14%) | 1,093 (13%) | 1,937 (15%) |
| *2010* | 19,131 (13%) | 7,148 (12%) | 9,000 (14%) | 1,063 (13%) | 1,920 (15%) |
| *2011* | 18,962 (13%) | 7,850 (13%) | 8,391 (13%) | 1,077 (13%) | 1,644 (13%) |
| *2012* | 18,781 (13%) | 7,727 (13%) | 8,475 (13%) | 1,002 (12%) | 1,577 (12%) |
| *2013* | 18,773 (13%) | 8,620 (14%) | 7,953 (12%) | 948 (12%) | 1,252 (10%) |
| *2014* | 13,893 (9%) | 6,467 (11%) | 5,851 (9%) | 701 (9%) | 874 (7%) |
|  |  |  |  |  |  |
| Reason for end of follow-up |  |  |  |  |  |
| *Day 42* | 147,264 (100%) | 60,646 (100%) | 65,550 (100%) | 8,129 (100%) | 12,939 (100%) |
| *Loss of enrollment* | 137 (0%) | 78 (0%) | 56 (0%) | 2 (0%) | 1 (0%) |
| *Death* | 13 (0%) | 7 (0%) | 6 (0%) | 0 (0%) | 0 (0%) |
|  |  |  |  |  |  |
| Delivery characteristics |  |  |  |  |  |
| *Severe maternal morbidity* | 1,359 (1%) | 569 (1%) | 589 (1%) | 89 (1%) | 112 (1%) |
| *Bilateral tubal ligation* | 5,096 (3%) | 764 (1%) | 3,348 (5%) | 139 (2%) | 845 (7%) |
| *Laceration* | 1,504 (1%) | 414 (1%) | 873 (1%) | 43 (0%) | 174 (1%) |
|  |  |  |  |  |  |
| Comorbidities and medication use |  |  |  |  |  |
| *Tobacco use* | 37,422 (25%) | 11,999 (20%) | 17,868 (27%) | 2,157 (27%) | 5,398 (42%) |
| *Pre-delivery opioid use* | 19,339 (13%) | 6,046 (10%) | 9,072 (14%) | 1,433 (18%) | 2,788 (22%) |
| *Benzodiazepine use* | 3,491 (2%) | 1,027 (2%) | 1,676 (3%) | 201 (2%) | 587 (5%) |
| *Stimulant use* | 7,104 (5%) | 1,874 (3%) | 3,635 (6%) | 431 (5%) | 1,164 (9%) |
| *Depression medication use* | 456 (0%) | 139 (0%) | 199 (0%) | 34 (0%) | 84 (1%) |
| *Antipsychotic use* | 822 (0%) | 292 (0%) | 358 (0%) | 59 (1%) | 113 (1%) |
| *Depression diagnosis* | 3,006 (2%) | 1,103 (2%) | 1,340 (2%) | 200 (2%) | 363 (3%) |
| *Anxiety diagnosis* | 1,546 (1%) | 570 (1%) | 675 (1%) | 103 (1%) | 198 (2%) |
| *Psychosis diagnosis* | 214 (0%) | 151 (0%) | 40 (0%) | 13 (0%) | 10 (0%) |
| *Bipolar diagnosis* | 1,888 (1%) | 754 (1%) | 786 (1%) | 123 (2%) | 225 (2%) |
| *NSAID contraindication* | 277 (0%) | 112 (0%) | 121 (0%) | 13 (0%) | 31 (0%) |
| *Abdominal pain* | 1,367 (1%) | 450 (1%) | 618 (1%) | 116 (1%) | 183 (1%) |
| *Dental pain* | 210 (0%) | 65 (0%) | 90 (0%) | 13 (0%) | 42 (0%) |
| *Genitourinary pain* | 1,041 (1%) | 421 (1%) | 443 (1%) | 65 (1%) | 112 (1%) |
| *Trauma diagnosis* | 4,212 (3%) | 1,578 (3%) | 1,859 (3%) | 279 (3%) | 496 (4%) |
| *Musculoskeletal pain* | 6,496 (4%) | 2,446 (4%) | 2,896 (4%) | 407 (5%) | 747 (6%) |
| *Back pain* | 8,676 (6%) | 3,020 (5%) | 4,015 (6%) | 545 (7%) | 1,096 (8%) |
| *Autoimmune pain* | 243 (0%) | 108 (0%) | 86 (0%) | 18 (0%) | 31 (0%) |
| *Arthritis pain* | 8,281 (6%) | 2,882 (5%) | 3,840 (6%) | 523 (6%) | 1,036 (8%) |
| *Neck pain* | 160 (0%) | 59 (0%) | 69 (0%) | 11 (0%) | 21 (0%) |
| *Neurological pain* | 391 (0%) | 146 (0%) | 162 (0%) | 30 (0%) | 53 (0%) |
| *Malignancy* | 2,052 (1%) | 680 (1%) | 1,059 (2%) | 112 (1%) | 201 (2%) |
| *Irritable bowel syndrome* | 719 (0%) | 311 (0%) | 288 (0%) | 50 (1%) | 70 (0%) |
| *Headache* | 3,945 (3%) | 1,494 (2%) | 1,699 (3%) | 266 (3%) | 486 (4%) |
| *Migraine* | 899 (1%) | 341 (0%) | 384 (0%) | 71 (1%) | 103 (1%) |
| *Pain not otherwise specified* | 1,229 (1%) | 486 (1%) | 525 (1%) | 85 (1%) | 133 (1%) |
| *Other pain condition* | 1,774 (1%) | 702 (1%) | 750 (1%) | 117 (1%) | 205 (2%) |

**Figure S1**. Graphical representation various models. Panels (A) and (B) illustrate the simple linear model in the absence and presence (respectively) of a spike effect. Panel (C) illustrates the case in which dose group is categorized (with zero included as a category). Panels (C) and (D) illustrate the natural cubic spline model in the absence and presence (respectively) of a spike effect. Panel (F) illustrates the “slab-and-spline” model that is piecewise constant over the low-dose range but otherwise shares the properties of a natural cubic spline. Of note, the models presented in panels (A) and (D) invoke the safe-dose assumption as no spike effect is present.

**Figure S2**. Characterization of dose distribution in each of two simulation scenarios.

**Figure S3**. Simulation results in the absence of a spike effect for dose scenario 1. The true dose-response curve is shown as a solid black line. The average estimate across simulations is depicted as a solid blue line. Bars representing (1.96) times the empirical standard error (i.e., the standard deviation of the estimates across simulations, shown in pink) and the average standard error (i.e., the average of the estimated standard errors within dose groups across simulation, shown in light blue) are also shown at a variety of dose values. Further, the estimate coverage as is presented as a function of dose as a solid red line, with a horizontal dash at 0.95 marking the target coverage threshold (secondary axis in the upper-right hand of each plot).

**Figure S4**. Simulation results in the absence of a spike effect for dose scenario 2. The true dose-response curve is shown as a solid black line. The average estimate across simulations is depicted as a solid blue line. Bars representing (1.96) times the empirical standard error (i.e., the standard deviation of the estimates across simulations, shown in pink) and the average standard error (i.e., the average of the estimated standard errors within dose groups across simulation, shown in light blue) are also shown at a variety of dose values. Further, the estimate coverage as is presented as a function of dose as a solid red line, with a horizontal dash at 0.95 marking the target coverage threshold (secondary axis in the upper-right hand of each plot).

**Figure S5**. Illustration with example code for slab effect. Panel (A) depicts a scatter plot of simulated data example, with estimate and pointwise 95% confidence band for predicted mean across levels of the exposure. Panel (B) presents the estimated and pointwise 95% confidence band for differences relative to zero-dos under a slab-effect model.

**Figure S6**. Panel (A) depicts the distribution of the total dose of opioids prescribed in the initial postpartum period (day 3 before delivery through day 4 after delivery), up through MME of 1500 (maximum initial MME: 2070]. Panel (B) depicts the distribution of the total dose of opioids prescribed in the subsequent postpartum period (day 5 through day 42 after delivery), up through MME of 1500 [maximum subsequent MME: 16800].


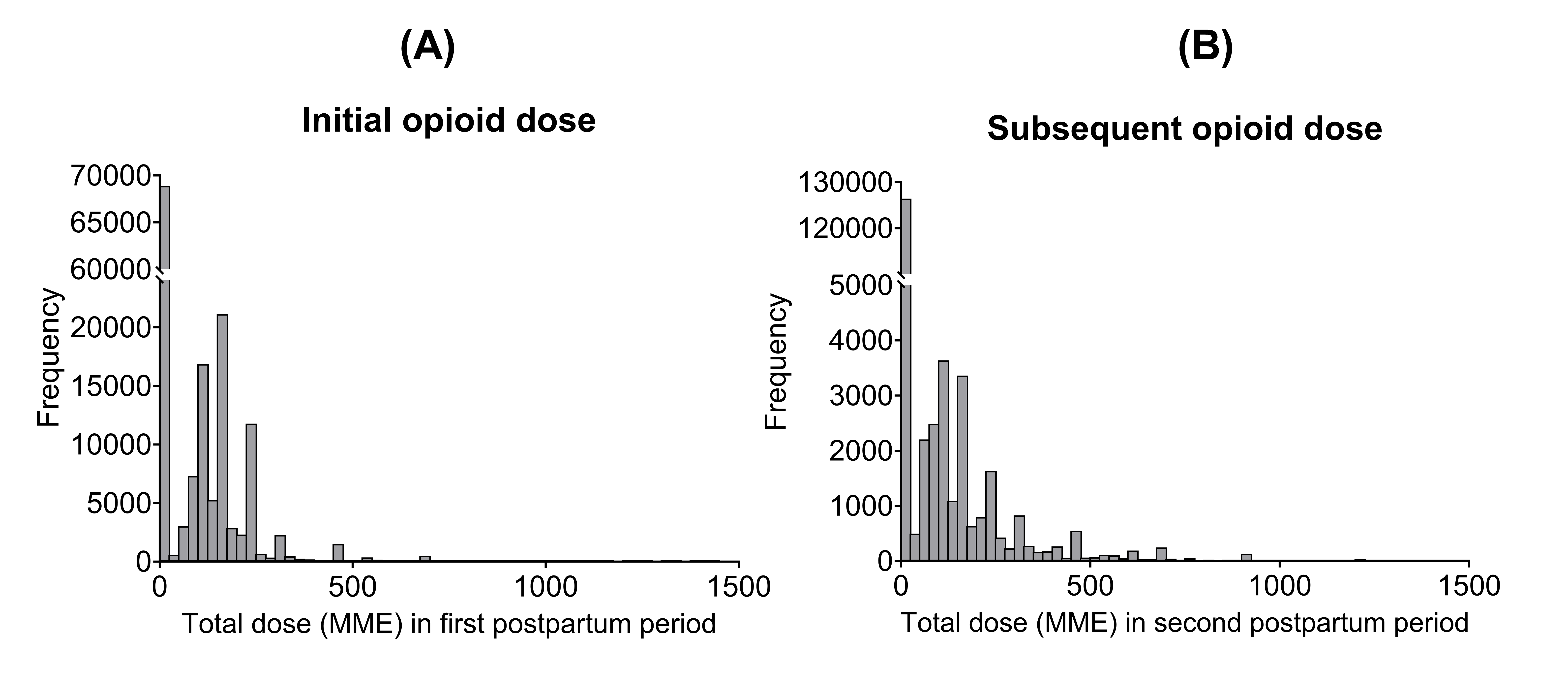

Supplement: Web_Material_kwae147 [file web_material_kwae147.docx]
